# 4-Bromo-2-[(*E*)-{[4-nitro-2-(trifluoro­meth­yl)phen­yl]imino}­meth­yl]phenol

**DOI:** 10.1107/S1600536812042262

**Published:** 2012-10-20

**Authors:** Mehmet Akkurt, Alan R. Kennedy, Shaaban K. Mohamed, Antar A. Abdelhamid, Gary J. Miller

**Affiliations:** a Department of Physics, Faculty of Sciences, Erciyes University, 38039 Kayseri, Turkey; b Department of Pure & Applied Chemistry, University of Strathclyde, 295 Cathedral Street, Glasgow G1 1XL, Scotland; c Chemistry and Environmental Division, Manchester Metropolitan University, Manchester, M1 5GD, England; d Analytical Sciences, Manchester Metropolitan University, Manchester, M1 5GD, England

## Abstract

Except two F atoms of the –CF_3_ group, the title compound, C_14_H_8_BrF_3_N_2_O_3_, has an almost planar conformation, the dihedral angle between the aromatic rings being 3.60 (16)°. The mol­ecule adopts the enol–imine tautomeric form, with an intra­molecular O—H⋯N hydrogen bond, which generates an *S*(6) ring motif. In the crystal, face-to-face π–π stacking [centroid–centroid distances = 3.669 (2) and 3.732 (2) Å] between the aromatic rings of the mol­ecules, which lie in sheets parallel to (202), help to establish the packing.

## Related literature

For the biological activity of fluorine-containing compounds, see: Blair *et al.* (2000 ▶); Chawla *et al.* (2012 ▶); Bella *et al.* (2004 ▶); Chandra & Kumar (2005 ▶); Yang *et al.* (2000 ▶). For the synthesis of a similar azomethine compound, see: Mohamed *et al.* (2012 ▶). For the graph-set analysis of hydrogen bonding, see: Bernstein *et al.* (1995 ▶).
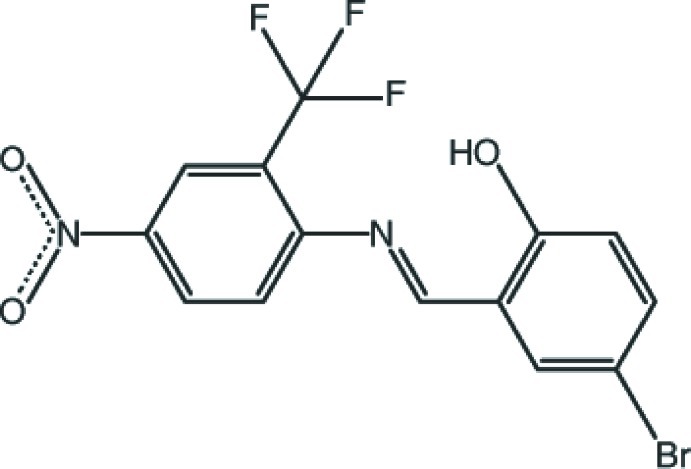


## Experimental

### 

#### Crystal data

C_14_H_8_BrF_3_N_2_O_3_
*M* *_r_* = 389.12
Monoclinic, 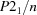

*a* = 7.3596 (5) Å
*b* = 16.4625 (10) Å
*c* = 11.2599 (6) Å
β = 94.955 (5)°
*V* = 1359.12 (14) Å^3^
*Z* = 4
Mo *K*α radiation
μ = 3.08 mm^−1^
*T* = 123 K
0.20 × 0.18 × 0.18 mm

#### Data collection

Oxford Diffraction Xcalibur Eos diffractometer
Absorption correction: multi-scan (*CrysAlis PRO*; Oxford Diffraction, 2010 ▶) *T*_min_ = 0.546, *T*_max_ = 0.575
6329 measured reflections
3149 independent reflections
2165 reflections with *I* > 2σ(*I*)
*R*_int_ = 0.043

#### Refinement

*R*[*F* ^2^ > 2σ(*F* ^2^)] = 0.050
*wR*(*F* ^2^) = 0.090
*S* = 1.02
3149 reflections
203 parameters
1 restraint
H-atom parameters constrained
Δρ_max_ = 0.51 e Å^−3^
Δρ_min_ = −0.59 e Å^−3^

### 

Data collection: *CrysAlis PRO* (Oxford Diffraction, 2010 ▶); cell refinement: *CrysAlis PRO*; data reduction: *CrysAlis PRO*; program(s) used to solve structure: *SHELXS97* (Sheldrick, 2008 ▶); program(s) used to refine structure: *SHELXL97* (Sheldrick, 2008 ▶); molecular graphics: *ORTEP-3 for Windows* (Farrugia, 1997 ▶); software used to prepare material for publication: *WinGX* (Farrugia, 1999 ▶) and *PLATON* (Spek, 2009 ▶).

## Supplementary Material

Crystal structure: contains datablock(s) global, I. DOI: 10.1107/S1600536812042262/xu5632sup1.cif

Structure factors: contains datablock(s) I. DOI: 10.1107/S1600536812042262/xu5632Isup2.hkl

Additional supplementary materials:  crystallographic information; 3D view; checkCIF report

## Figures and Tables

**Table 1:** Hydrogen-bond geometry (Å, °)

*D*—H⋯*A*	*D*—H	H⋯*A*	*D*⋯*A*	*D*—H⋯*A*
O3—H1*O*⋯N1	0.84	1.88	2.623 (4)	146

**Fig. 1.**
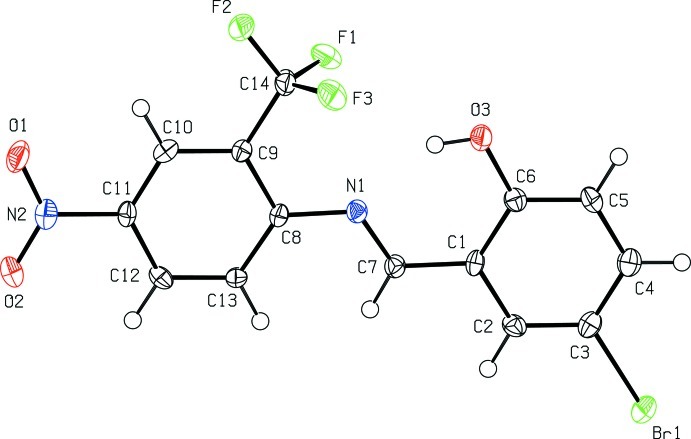
The title compound with the atom numbering scheme. Displacement ellipsoids for non-H atoms are drawn at the 50% probability level.

**Fig. 2.**
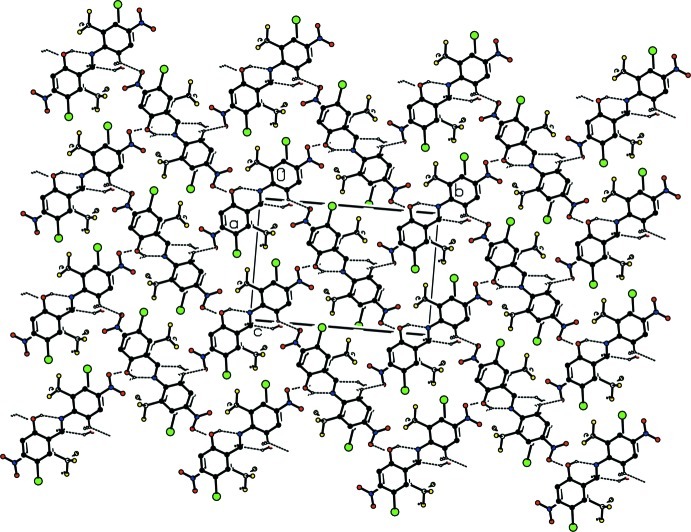
View of the packing and hydrogen bonding of (I) down a axis. H atoms not involved in hydrogen bonding have been omitted for clarity.

**Fig. 3.**
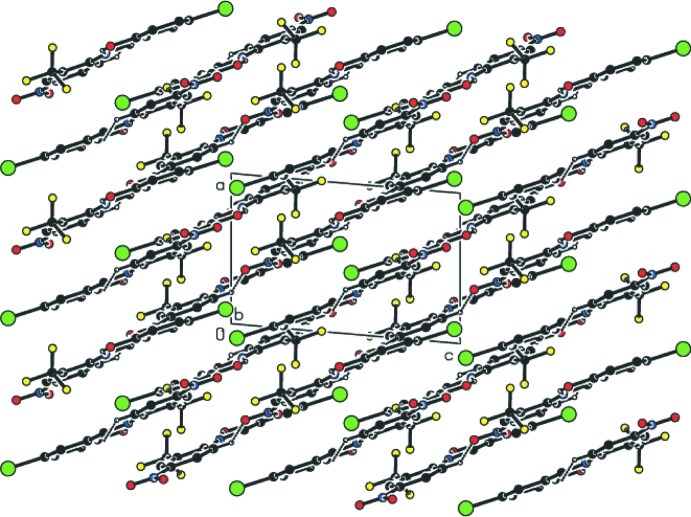
View of the packing and hydrogen bonding of (I) down b axis. H atoms not involved in hydrogen bonding have been omitted for clarity.

## supplementary crystallographic information

### Comment

A great number of Schiff base complexes with metals have provoked wide interest because they possess a diverse spectrum of biological and pharmaceutical activities, such as antitumor and anti-oxidative activities, as well as the inhibition of lipid peroxidation (Bella *et al.*, 2004; Chandra & Kumar, 2005; Yang *et al.*, 2000). Fluorine can dramatically change the properties of biologically active compounds and can influence the metabolism and distribution of drug molecules in the body (Blair *et al.*, 2000). Recently, SAR studies revealed that the presence of a fluoro group had a marked influence on the antibacterial activity (Chawla *et al.*, 2012). Such facts and further to our studies on synthesis of bio-active molecules we herein report the synthesis and crystal structure of a new potential bio-active fluorinated azomethine compound (I).

Fig. 1 shows the title compound (I) with the enol-imine tautomeric form, which has an intramolecular O— H···N hydrogen bond forming an S(6) motif (Bernstein *et al.*, 1995; Table 1). The C6—O3 single bond of 1.354 (4) Å and the C7═N1 double bond of 1.293 (4) Å verify the enol-imine form. These distances and the values of the other geometric parameters are in the normal range and are comparable with those of a similar compound reported previously (Mohamed *et al.*, 2012). The two aromatic rings (C1–C6 and C8–C13) make a dihedral angle of 3.60 (16)° with each other. The C1—C7—N1—C8, O1—N2—C11—C10, O2—N2—C11—C10, C14–C9—C8—N1, C8—N1—C7—C1, C7—C1—C6—O3 and C1—C2—C3—Br1 torsion angles are 178.1 (3), 2.4 (4), 175.8 (3), -1.4 (5), -178.1 (3), 0.0 (6) and 179.2 (3) °, respectively. Therefore, the whole molecule of (I), except the F1 and F3 atoms of the –CF_3_ group, is almostly planar.

The crystal structure is stabilized by face-to-face π-π stacking interactions [*Cg*1···*Cg*2(1 - *x*, -*y*, 2 - *z*) = 3.669 (2) Å and *Cg*1···*Cg*2(2 - *x*, -*y*, 2 - *z*) = 3.732 (2) Å] between the *Cg*1 and *Cg*2 centroids of the C1–C6 and C8–C13 aromatic rings of the molecules to form two-dimensional sheets parallel to the (202) plane (Fig. 2 & Fig. 3).

### Experimental

The title compound was unexpectedly obtained from a three component reaction of 0.01 mol 4-nitro-2-(trifluoromethyl)aniline, 0.01 mol 5-bromosalicyaldehyde and 0.01 mol 5-phenyl-1,3-cyclohexanedione in 50 ml ethanol. The reaction mixture was refluxed for 7 h at 350 K. The solid product that obtained on cooling was filtered off, washed with cold ethanol and dried. The crude product was recrystallized from a mixture of ethanol and acetone (10:1 vv) to afford a good quality crystals suitable for X-ray difraction after two days of slow evaporation at room temperature. [Yield 83%; Mp. 511 K].

### Refinement

All H atoms were positioned geometrically [C—H = 0.95 Å and O—H = 0.84 Å] and refined as riding with *U*_iso_(H) = 1.2*U*_eq_(C) for aromatic H and *U*_iso_(H) = 1.5*U*_eq_(O) for hydroxyl H. The components of anisotropic displacement for N1 and C7 atoms were made equal using the EADP constraint.

### Crystal data

**Table:**

C_14_H_8_BrF_3_N_2_O_3_	*F*(000) = 768
*M**_r_* = 389.12	*D*_x_ = 1.902 Mg m^−^^3^
Monoclinic, *P*2_1_/*n*	Mo *K*α radiation, λ = 0.71073 Å
Hall symbol: -P 2yn	Cell parameters from 1836 reflections
*a* = 7.3596 (5) Å	θ = 3.2–29.0°
*b* = 16.4625 (10) Å	µ = 3.08 mm^−^^1^
*c* = 11.2599 (6) Å	*T* = 123 K
β = 94.955 (5)°	Cut rod, yellow
*V* = 1359.12 (14) Å^3^	0.20 × 0.18 × 0.18 mm
*Z* = 4	

### Data collection

**Table:**

Oxford Diffraction Xcalibur Eos diffractometer	3149 independent reflections
Radiation source: Enhance (Mo) X-ray Source	2165 reflections with *I* > 2σ(*I*)
Graphite monochromator	*R*_int_ = 0.043
Detector resolution: 16.0727 pixels mm^-1^	θ_max_ = 29.0°, θ_min_ = 3.2°
ω scans	*h* = −9→8
Absorption correction: multi-scan (*CrysAlis PRO*; Oxford Diffraction, 2010)	*k* = −18→22
*T*_min_ = 0.546, *T*_max_ = 0.575	*l* = −14→15
6329 measured reflections	

### Refinement

**Table:**

Refinement on *F*^2^	Primary atom site location: structure-invariant direct methods
Least-squares matrix: full	Secondary atom site location: difference Fourier map
*R*[*F*^2^ > 2σ(*F*^2^)] = 0.050	Hydrogen site location: inferred from neighbouring sites
*wR*(*F*^2^) = 0.090	H-atom parameters constrained
*S* = 1.02	*w* = 1/[σ^2^(*F*_o_^2^) + (0.0233*P*)^2^] where *P* = (*F*_o_^2^ + 2*F*_c_^2^)/3
3149 reflections	(Δ/σ)_max_ < 0.001
203 parameters	Δρ_max_ = 0.51 e Å^−^^3^
1 restraint	Δρ_min_ = −0.59 e Å^−^^3^

### Special details

**Table:**

Geometry. Bond distances, angles *etc*. have been calculated using the rounded fractional coordinates. All su's are estimated from the variances of the (full) variance-covariance matrix. The cell e.s.d.'s are taken into account in the estimation of distances, angles and torsion angles
Refinement. Refinement on *F*^2^ for ALL reflections except those flagged by the user for potential systematic errors. Weighted *R*-factors *wR* and all goodnesses of fit *S* are based on *F*^2^, conventional *R*-factors *R* are based on *F*, with *F* set to zero for negative *F*^2^. The observed criterion of *F*^2^ > σ(*F*^2^) is used only for calculating -*R*-factor-obs *etc*. and is not relevant to the choice of reflections for refinement. *R*-factors based on *F*^2^ are statistically about twice as large as those based on *F*, and *R*-factors based on ALL data will be even larger.

### Fractional atomic coordinates and isotropic or equivalent isotropic displacement parameters (Å^2^)

**Table:**

	*x*	*y*	*z*	*U*_iso_*/*U*_eq_	
Br1	0.40760 (6)	0.10935 (3)	0.52512 (3)	0.0305 (2)	
F1	0.7671 (3)	0.08414 (14)	1.28090 (17)	0.0313 (8)	
F2	1.0022 (3)	0.05148 (15)	1.39501 (17)	0.0404 (9)	
F3	1.0339 (3)	0.09587 (14)	1.21958 (18)	0.0321 (8)	
O1	1.1326 (3)	−0.22498 (18)	1.4389 (2)	0.0299 (9)	
O2	1.0746 (4)	−0.31205 (17)	1.2960 (2)	0.0286 (9)	
O3	0.7242 (4)	0.17073 (17)	1.02757 (19)	0.0262 (9)	
N1	0.7788 (4)	0.01384 (19)	1.0499 (2)	0.0168 (7)	
N2	1.0692 (4)	−0.2434 (2)	1.3380 (3)	0.0231 (11)	
C1	0.6384 (5)	0.0741 (2)	0.8725 (3)	0.0160 (11)	
C2	0.5635 (4)	0.0613 (2)	0.7551 (3)	0.0185 (11)	
C3	0.5065 (5)	0.1256 (3)	0.6850 (3)	0.0192 (11)	
C4	0.5184 (5)	0.2040 (3)	0.7277 (3)	0.0220 (12)	
C5	0.5917 (5)	0.2182 (2)	0.8437 (3)	0.0210 (12)	
C6	0.6520 (5)	0.1540 (2)	0.9156 (3)	0.0176 (11)	
C7	0.6997 (4)	0.0045 (2)	0.9437 (3)	0.0168 (7)	
C8	0.8434 (4)	−0.0537 (2)	1.1180 (3)	0.0145 (11)	
C9	0.9215 (5)	−0.0375 (2)	1.2358 (3)	0.0158 (11)	
C10	0.9916 (4)	−0.1002 (2)	1.3070 (3)	0.0185 (11)	
C11	0.9869 (4)	−0.1777 (2)	1.2628 (3)	0.0165 (11)	
C12	0.9119 (5)	−0.1958 (2)	1.1491 (3)	0.0194 (12)	
C13	0.8421 (5)	−0.1338 (2)	1.0781 (3)	0.0190 (11)	
C14	0.9282 (5)	0.0480 (3)	1.2828 (3)	0.0225 (12)	
H1O	0.75720	0.12730	1.06220	0.0390*	
H2	0.55230	0.00760	0.72440	0.0220*	
H4	0.47670	0.24810	0.67820	0.0260*	
H5	0.60030	0.27220	0.87360	0.0250*	
H7	0.68160	−0.04870	0.91210	0.0200*	
H10	1.04260	−0.08970	1.38590	0.0220*	
H12	0.90880	−0.25020	1.12080	0.0230*	
H13	0.79150	−0.14570	0.99960	0.0230*	

### Atomic displacement parameters (Å^2^)

**Table:**

	*U*^11^	*U*^22^	*U*^33^	*U*^12^	*U*^13^	*U*^23^
Br1	0.0418 (3)	0.0288 (3)	0.0189 (2)	0.0038 (2)	−0.0083 (2)	0.0014 (2)
F1	0.0389 (14)	0.0225 (14)	0.0323 (13)	0.0074 (12)	0.0023 (10)	−0.0076 (11)
F2	0.0708 (18)	0.0249 (16)	0.0215 (12)	0.0036 (15)	−0.0195 (11)	−0.0031 (11)
F3	0.0407 (14)	0.0180 (14)	0.0370 (13)	−0.0091 (12)	−0.0002 (10)	−0.0010 (11)
O1	0.0367 (17)	0.0287 (18)	0.0221 (14)	−0.0014 (15)	−0.0098 (11)	0.0078 (14)
O2	0.0364 (17)	0.0168 (17)	0.0325 (15)	0.0032 (15)	0.0019 (12)	0.0047 (13)
O3	0.0387 (17)	0.0174 (16)	0.0206 (14)	0.0006 (15)	−0.0082 (11)	0.0014 (12)
N1	0.0190 (12)	0.0145 (13)	0.0168 (11)	−0.0024 (11)	0.0008 (9)	0.0019 (11)
N2	0.0215 (18)	0.023 (2)	0.0249 (18)	0.0009 (17)	0.0021 (13)	0.0074 (16)
C1	0.0146 (19)	0.017 (2)	0.0164 (18)	−0.0012 (17)	0.0015 (14)	0.0063 (17)
C2	0.0190 (19)	0.015 (2)	0.021 (2)	−0.0019 (18)	−0.0007 (14)	−0.0029 (17)
C3	0.0156 (19)	0.024 (2)	0.0178 (19)	−0.0020 (18)	−0.0004 (14)	0.0031 (17)
C4	0.020 (2)	0.023 (2)	0.023 (2)	−0.0011 (19)	0.0022 (15)	0.0039 (18)
C5	0.025 (2)	0.013 (2)	0.025 (2)	0.0003 (18)	0.0022 (15)	0.0016 (17)
C6	0.0170 (19)	0.016 (2)	0.0198 (19)	−0.0017 (18)	0.0014 (14)	0.0005 (17)
C7	0.0190 (12)	0.0145 (13)	0.0168 (11)	−0.0024 (11)	0.0008 (9)	0.0019 (11)
C8	0.0136 (18)	0.016 (2)	0.0139 (18)	0.0001 (17)	0.0015 (13)	0.0036 (16)
C9	0.0169 (19)	0.016 (2)	0.0144 (18)	−0.0003 (17)	0.0015 (14)	0.0020 (16)
C10	0.0168 (19)	0.025 (2)	0.0136 (18)	−0.0014 (19)	0.0009 (13)	0.0002 (17)
C11	0.0138 (19)	0.018 (2)	0.0177 (18)	0.0029 (17)	0.0018 (14)	0.0057 (17)
C12	0.022 (2)	0.012 (2)	0.024 (2)	−0.0020 (18)	0.0015 (15)	−0.0028 (17)
C13	0.028 (2)	0.014 (2)	0.0139 (18)	−0.0011 (18)	−0.0037 (15)	−0.0018 (16)
C14	0.029 (2)	0.017 (2)	0.020 (2)	−0.001 (2)	−0.0071 (16)	0.0025 (17)

### Geometric parameters (Å, º)

**Table:**

Br1—C3	1.902 (3)	C4—C5	1.389 (5)
F1—C14	1.325 (5)	C5—C6	1.381 (5)
F2—C14	1.333 (4)	C8—C13	1.393 (5)
F3—C14	1.352 (5)	C8—C9	1.425 (5)
O1—N2	1.228 (4)	C9—C10	1.379 (5)
O2—N2	1.227 (4)	C9—C14	1.503 (6)
O3—C6	1.354 (4)	C10—C11	1.369 (5)
O3—H1O	0.8400	C11—C12	1.382 (5)
N1—C7	1.293 (4)	C12—C13	1.369 (5)
N1—C8	1.410 (4)	C2—H2	0.9500
N2—C11	1.471 (5)	C4—H4	0.9500
C1—C6	1.403 (5)	C5—H5	0.9500
C1—C7	1.448 (5)	C7—H7	0.9500
C1—C2	1.404 (5)	C10—H10	0.9500
C2—C3	1.365 (5)	C12—H12	0.9500
C3—C4	1.378 (7)	C13—H13	0.9500
			
C6—O3—H1O	109.00	N2—C11—C10	118.7 (3)
C7—N1—C8	120.9 (3)	C10—C11—C12	122.2 (3)
O1—N2—C11	117.1 (3)	N2—C11—C12	119.1 (3)
O2—N2—C11	118.7 (3)	C11—C12—C13	118.7 (3)
O1—N2—O2	124.2 (3)	C8—C13—C12	121.7 (3)
C2—C1—C7	118.8 (3)	F1—C14—F3	106.5 (3)
C6—C1—C7	122.8 (3)	F1—C14—C9	114.4 (3)
C2—C1—C6	118.5 (3)	F1—C14—F2	106.7 (3)
C1—C2—C3	120.3 (3)	F2—C14—C9	111.9 (3)
Br1—C3—C2	120.8 (3)	F3—C14—C9	111.3 (3)
Br1—C3—C4	118.0 (3)	F2—C14—F3	105.5 (3)
C2—C3—C4	121.2 (3)	C1—C2—H2	120.00
C3—C4—C5	119.6 (4)	C3—C2—H2	120.00
C4—C5—C6	120.1 (3)	C3—C4—H4	120.00
O3—C6—C5	118.1 (3)	C5—C4—H4	120.00
C1—C6—C5	120.3 (3)	C4—C5—H5	120.00
O3—C6—C1	121.6 (3)	C6—C5—H5	120.00
N1—C7—C1	120.8 (3)	N1—C7—H7	120.00
N1—C8—C13	125.4 (3)	C1—C7—H7	120.00
C9—C8—C13	117.9 (3)	C9—C10—H10	120.00
N1—C8—C9	116.7 (3)	C11—C10—H10	120.00
C8—C9—C14	120.1 (3)	C11—C12—H12	121.00
C10—C9—C14	119.8 (3)	C13—C12—H12	121.00
C8—C9—C10	120.1 (3)	C8—C13—H13	119.00
C9—C10—C11	119.3 (3)	C12—C13—H13	119.00
			
C8—N1—C7—C1	−178.1 (3)	C4—C5—C6—C1	−0.5 (6)
C7—N1—C8—C9	−177.4 (3)	N1—C8—C9—C10	−178.5 (3)
C7—N1—C8—C13	4.8 (5)	N1—C8—C9—C14	1.4 (5)
O1—N2—C11—C10	2.4 (4)	C13—C8—C9—C10	−0.5 (5)
O1—N2—C11—C12	−179.2 (3)	C13—C8—C9—C14	179.3 (3)
O2—N2—C11—C10	−175.8 (3)	N1—C8—C13—C12	178.3 (3)
O2—N2—C11—C12	2.5 (5)	C9—C8—C13—C12	0.5 (5)
C6—C1—C2—C3	0.3 (5)	C8—C9—C10—C11	0.7 (5)
C7—C1—C2—C3	−179.2 (3)	C14—C9—C10—C11	−179.2 (3)
C2—C1—C6—O3	−179.5 (3)	C8—C9—C14—F1	57.2 (4)
C2—C1—C6—C5	0.4 (5)	C8—C9—C14—F2	178.7 (3)
C7—C1—C6—O3	0.0 (6)	C8—C9—C14—F3	−63.6 (4)
C7—C1—C6—C5	179.9 (3)	C10—C9—C14—F1	−122.9 (3)
C2—C1—C7—N1	176.1 (3)	C10—C9—C14—F2	−1.5 (5)
C6—C1—C7—N1	−3.4 (5)	C10—C9—C14—F3	116.3 (4)
C1—C2—C3—Br1	179.2 (3)	C9—C10—C11—N2	177.4 (3)
C1—C2—C3—C4	−0.9 (5)	C9—C10—C11—C12	−0.8 (5)
Br1—C3—C4—C5	−179.3 (3)	N2—C11—C12—C13	−177.5 (3)
C2—C3—C4—C5	0.8 (6)	C10—C11—C12—C13	0.8 (5)
C3—C4—C5—C6	−0.1 (6)	C11—C12—C13—C8	−0.6 (5)
C4—C5—C6—O3	179.3 (3)		

### Hydrogen-bond geometry (Å, º)

**Table:**

*D*—H···*A*	*D*—H	H···*A*	*D*···*A*	*D*—H···*A*
O3—H1*O*···N1	0.84	1.88	2.623 (4)	146
C10—H10···F2	0.95	2.35	2.685 (4)	100
C13—H13···O1^i^	0.95	2.50	3.134 (4)	125

Symmetry code: (i) *x*−1/2, −*y*−1/2, *z*−1/2.
